# Ancient DNA Suggests Dwarf and ‘Giant’ Emu Are Conspecific

**DOI:** 10.1371/journal.pone.0018728

**Published:** 2011-04-11

**Authors:** Tim H. Heupink, Leon Huynen, David M. Lambert

**Affiliations:** Griffith School of Environment and School of Biomolecular and Physical Sciences, Griffith University, Nathan, Australia; Smithsonian Institution National Zoological Park, United States of America

## Abstract

**Background:**

The King Island Emu (*Dromaius ater*) of Australia is one of several extinct emu taxa whose taxonomic relationship to the modern Emu (*D. novaehollandiae*) is unclear. King Island Emu were mainly distinguished by their much smaller size and a reported darker colour compared to modern Emu.

**Methodology and Results:**

We investigated the evolutionary relationships between the King Island and modern Emu by the recovery of both nuclear and mitochondrial DNA sequences from sub-fossil remains. The complete mitochondrial control (1,094 bp) and cytochrome *c* oxidase subunit I (COI) region (1,544 bp), as well as a region of the melanocortin 1 receptor gene (57 bp) were sequenced using a multiplex PCR approach. The results show that haplotypes for King Island Emu fall within the diversity of modern Emu.

**Conclusions:**

These data show the close relationship of these emu when compared to other congeneric bird species and indicate that the King Island and modern Emu share a recent common ancestor. King Island emu possibly underwent insular dwarfism as a result of phenotypic plasticity. The close relationship between the King Island and the modern Emu suggests it is most appropriate that the former should be considered a subspecies of the latter. Although both taxa show a close genetic relationship they differ drastically in size. This study also suggests that rates of morphological and neutral molecular evolution are decoupled.

## Introduction

**Figure 1:**
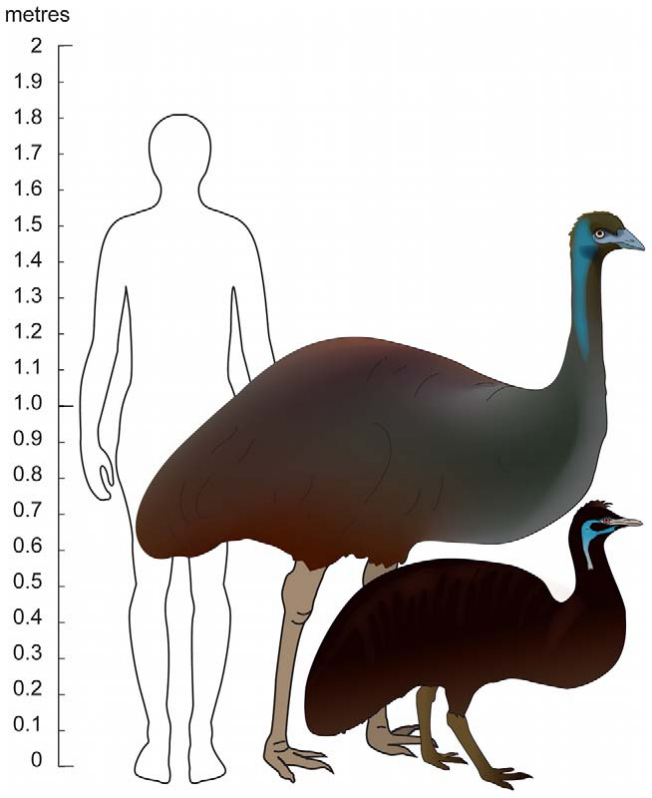
Modern and extinct emu. The modern Emu (centre) and King Island Emu (right) with human outline shown approximately to scale. Apart from obvious size differences, there were reports of colour differences between these emu taxa.

**Figure 2:**
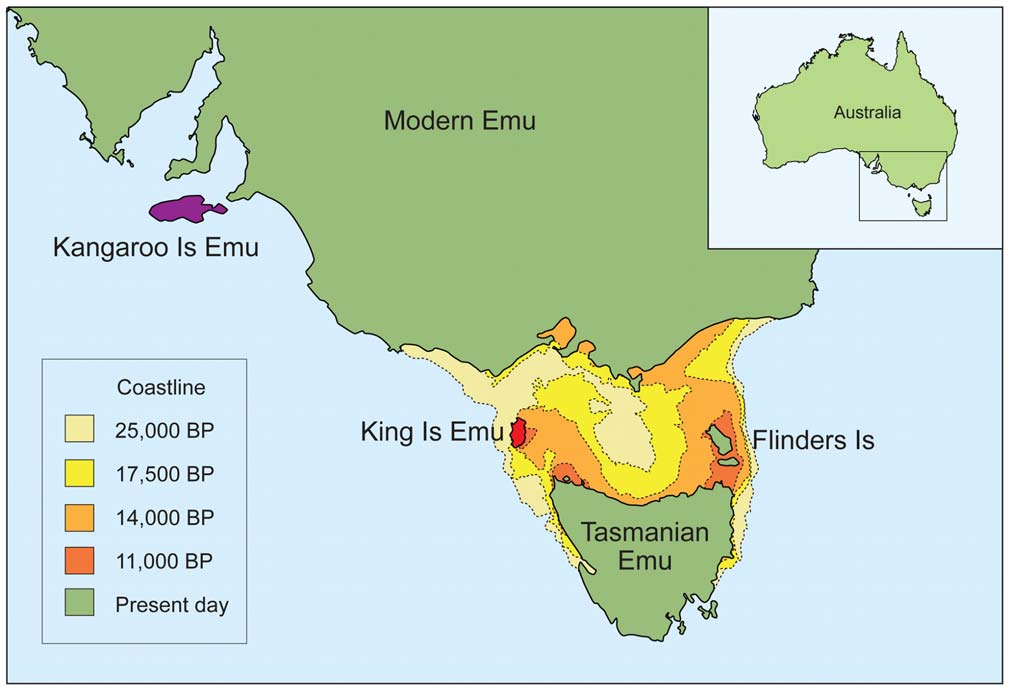
Geographic distribution of emu taxa and historic shoreline reconstructions around Tasmania. Modern Emu are currently found throughout mainland Australia. Extinct emu taxa were restricted to their respective islands: the Kangaroo Island Emu (purple), the King Island Emu (red) and the Tasmanian Emu. Twenty-five thousand years ago Tasmania, Flinders and King Island were connected to mainland Australia. Approximately 17,500 years ago King Island lost its direct connection with mainland Australia. By 14,000 years ago Tasmania, Flinders and King Island started to disconnect from the mainland, but were still connected to each other. By 11,000 years ago King Island was isolated from Tasmania, while the Tasmania was still connected to Flinders Island. Presently Tasmania, Flinders, King and Kangaroo Island are all isolated and disconnected from mainland Australia (modified from [23]).

During the Late Quaternary Australia's largest bird the Emu (*Dromaius novaehollandiae*) had several, often smaller relatives living on a number of offshore islands of mainland Australia. These included taxa found on Kangaroo Island *(D. baudinianus)*, King Island *(D. ater)* and Tasmania *(D. n. diemenensis*), all of which are now extinct. The smallest taxon, the King Island Emu, was confined to a small island situated in the Bass Strait between Tasmania and Victoria, approximately 100 km from both coasts (Figures 1 and 2). King Island was once part of the land bridge which connected Tasmania and mainland Australia, but rising sea levels following the last glacial maximum eventually isolated the island. The King Island Emu was first mentioned in January 1802 in exploration surveys of King Island, which described ‘woods full’ of emu and other animals [1], soon after English sealers settled on the island because of the abundance of elephant seals. In December 1802 Péron, a French naturalist who was part of Baudin's expedition, visited the island and was the last person to record descriptions of the King Island Emu [2]. The little we know today about the King Island Emu stems from interviews Péron conducted with the sealers. The emu was described as a small form and “quite black” compared with the mainland species. Soon after the visit by Péron the King Island Emu went extinct. The interviews with the sealers suggested why this bird did not survive for long. Péron described how dogs were purpose-trained to hunt down emu and a variety of cooking recipes are mentioned; one of the sealers even claimed to have killed no fewer than 300 emu. Today we know that several King Island Emu specimens were sent to France as part of Baudin's expedition [3]–[5], several of which survive as specimens in museums throughout Europe today.

Initially there was confusion regarding the taxonomic status and geographic origin of the King Island Emu, particularly with respect to their relationship to Kangaroo Island Emu, which were also transported to France as part of the same expedition. The expeditions logbooks failed to clearly state where and when dwarf emu individuals were collected. This led to both taxa being interpreted as a single taxon and that it originated from Kangaroo Island. More recent finds of sub-fossil material and subsequent studies on King and Kangaroo Island Emu confirm their separate geographic origin and distinct morphology. There are few morphological differences that distinguish dwarf emu taxa from modern Emu besides their size, but all three taxa are now nevertheless considered separate species [5]–[9]. The remains of the Tasmanian Emu are scarce. There are suggestions this bird was slightly smaller than the modern Emu, but in conflict, other evidence (including descriptions of Pleistocene remains) indicates that both are similar in size. The Tasmanian Emu has to date, been considered a subspecies of the modern Emu. This is likely to continue until more conclusive evidence clarifies this matter. Fossil emu from mainland Australia display a more “average” range of sizes between that of the dwarf and modern taxa [10].

To investigate the relationship between the modern Emu and the King Island Emu we characterised the complete mitochondrial control region and cytochrome *c* oxidase subunit I (COI) as well as part of the nuclear encoded melanocortin 1 receptor (MC1R) gene. In contrast to previous unsuccessful attempts to isolate DNA from King Island Emu [11], we used a multiplex PCR approach to amplify these loci from sub-fossil King Island Emu remains [12], and report the first ancient DNA sequences recovered for this taxon.

## Results and Discussion

We recovered nucleotide DNA sequences of the complete mitochondrial control and COI regions (1,094 and 1,544 bp respectively) from four King Island Emu specimens (KI01-04), in addition to a MC1R (57 bp) fragment for two of these (KI01-02). A fifth specimen did yield amplification products for the control and COI regions but was excluded from further analyses due to excessive molecular damage including fragmentation and type 2 miscoding lesions [13]. Each recovered sequence showed some signs of molecular damage in the form of DNA fragmentation and type 2 miscoding lesions to a lesser extent, indicating authentic ancient DNA. DNA was extracted in a dedicated ancient DNA laboratory and a control region and COI amplicon were independently replicated for each of two specimens at a separate ancient DNA facility. The independent replication showed identical sequences, thereby ruling out laboratory contamination from PCR products. However there is the unlikely possibility that all four King Island Emu specimens were contaminated by modern Emu specimens beforehand, although the overlapping multiplex approach and observed molecular damage (fragmentation and miscoding lesions) make this scenario extremely unlikely. The same loci were recovered from an additional eighteen modern Emu blood samples from Emu farms in Medina, Western Australia and Palmerston North, New Zealand (16 and 2 samples respectively), these farmed emu represent varying origins from the wild population of modern Emu.

The recovered King Island Emu MC1R fragments were identical to those of modern Emu and interestingly did not display a SNP most commonly associated with melanism in birds [14], [15]. This does not necessarily indicate that the modern Emu and the supposedly quite black King Island Emu shared the same plumage colour Other genetic or non-genetic factors might be responsible for the reported difference in plumage colour [16]. However, the fact that this likely cause of darker plumage coloration in birds is not detected in the King Island Emu sequences brings into question the validity of this taxonomic trait.

**Figure 3:**
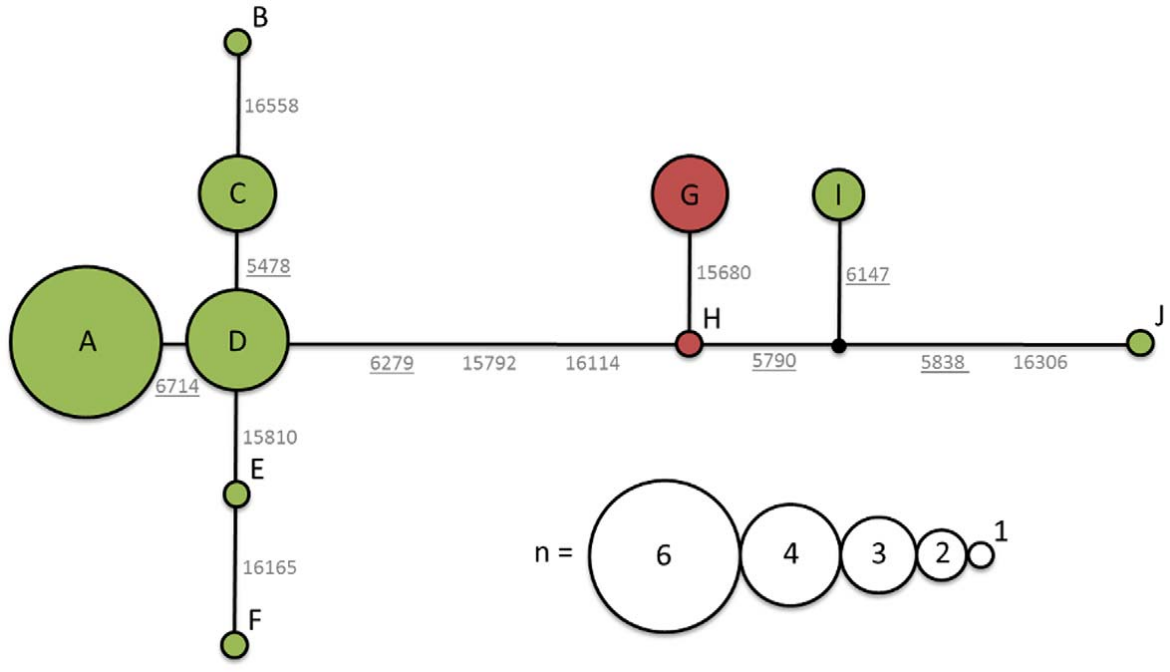
Haplotype network for modern Emu (green) and King Island Emu (red). Concatenated haplotypes of control and COI regions totalling 2638bp each. The black circle indicates a hypothetical haplotype, the distance between each neighbouring haplotype corresponds to the number of substitutions that separate them. Numbers correspond to positions in the mitochondrial genome as mentioned for Table 1, underlined numbers represent substitutions that occurred in the cytochrome *c* oxidase subunit I gene as supposed to the control region.

The control and COI regions recovered for both taxa show very little diversity, only seven and six sites respectively are polymorphic in alignments including the modern Emu mitochondrial genome reference sequence (Table 1). The sequences show no individual sites that fully discriminate both taxa, the King Island Emu sequences group phylogenetically with three modern Emu (AU01, NZ01 and NZ02) that share several segregating sites when compared to other modern Emu (two in the control and one in the COI region) (Figure 3). In order to confirm its authenticity the haplotype for modern Emu specimen AU01 has been replicated using several independent amplifications, including long range PCR to avoid nuclear copies and contamination.

**Table 1:** Sequence alignment and haplotype assignments.

		COI						Control region						
Sample	Haplo-type	5478	5790	5838	6147	6279	6714	15680	15792	15810	16114	16165	16306	16558
REF	F	C	C	T	T	C	T	T	C	C	G	A	T	A
AU01	J	.	T	C	.	T	.	.	T	T	A	C	C	.
AU02	D	.	.	.	.	.	.	.	.	T	.	C	.	.
AU03	C	A	.	.	.	.	.	.	.	T	.	C	.	.
AU04	D	.	.	.	.	.	.	.	.	T	.	C	.	.
AU05	A	.	.	.	.	.	C	.	.	T	.	C	.	.
AU06	C	A	.	.	.	.	.	.	.	T	.	C	.	.
AU07	D	.	.	.	.	.	.	.	.	T	.	C	.	.
AU08	A	.	.	.	.	.	C	.	.	T	.	C	.	.
AU09	B	A	.	.	.	.	.	.	.	T	.	C	.	C
AU10	D	.	.	.	.	.	.	.	.	T	.	C	.	.
AU11	A	.	.	.	.	.	C	.	.	T	.	C	.	.
AU12	A	.	.	.	.	.	C	.	.	T	.	C	.	.
AU13	C	A	.	.	.	.	.	.	.	T	.	C	.	.
AU14	A	.	.	.	.	.	C	.	.	T	.	C	.	.
AU15	E	.	.	.	.	.	.	.	.	.	.	C	.	.
AU16	A	.	.	.	.	.	C	.	.	T	.	C	.	.
NZ01	I	.	T	.	C	T	.	.	T	T	A	C	.	.
NZ02	I	.	T	.	C	T	.	.	T	T	A	C	.	.
KI01	G	.	.	.	.	T	.	C	T	T	A	C	.	.
KI02	H	.	.	.	.	T	.	.	T	T	A	C	.	.
KI03	G	.	.	.	.	T	.	C	T	T	A	C	.	.
KI04	G	.	.	.	.	T	.	C	T	T	A	C	.	.

Haplotype assignments refer to Figure 3. Numbers refer to position in Genbank reference sequence NC_002784.1. A point (.) refers to the same base as the reference sequence. Abbreviations are: REF – reference sequence, AU – Australian farmed, NZ – New Zealand farmed, KI – King Island Emu, COI – cytochrome *c* oxidase subunit I.

Although the King Island Emu display unique haplotypes for both the control and the COI regions, they fall within the diversity of modern Emu for both regions. This, in combination with the low control region and COI diversity, suggests that future studies may identify King Island Emu specific haplotypes in modern Emu. Hence this study would suggest that research aiming to distinguish both taxa using DNA should not be limited to the control or COI regions. Perhaps more highly variable nuclear sequences, like those often used in population studies (e.g. microsatellites or Major Histocompatibility Complex), may be better able to distinguish these taxa.

The sequence data recovered from both mitochondrial DNA regions indicate that the modern and the King Island Emu are very closely related. The control and COI regions of the King Island Emu fall within the diversity of modern Emu, showing the latter is a paraphyletic taxon. The low diversity in the sequences recovered for both taxa however indicates that incomplete lineage sorting is a likely cause for this pattern, in particular the processes involved in divergence of peripheral isolates as a result of founder effects [17], [18]. Both taxa show a very close paraphyletic relationship, the maximum distance between any King Island and modern Emu control and COI region haplotype is 0.46 and 0.13% (5 and 2 substitutions), respectively. The average pairwise distance for the control region between congeneric species has been reported 8.11% (ranging 0.54–26.24%) within a selection of bird genera [19]. This corresponds to 89 (ranging 6–287) substitutions for the control region length sequenced here. Nearest-neighbour distances between a large set of North American bird species' COI regions average 4.3% (ranging 0–14.18%). In contrast, the mean intraspecific distances for the same dataset average 0.23% (ranging 0–1.59%) [20]. The former corresponds to 66 (ranging 0–219) substitutions and the latter corresponds to 4 (ranging 0–25) substitutions for the COI region length sequenced in this study.

A small number of control and COI regions have been characterised for other ratites, mean pairwise differences among species from the same genus for the control region are (all excluding gaps): Kiwi (*Apteryx sp.*) 2.36, 8.22 and 8.65% (33, 115 & 121 sites, respectively, for 3 species totalling 3 sequences with an aligned length of 1399 basepairs), Rhea *(Rhea sp.)* 9.59% (116 sites, 2 species, 3 sequences, 1210 basepairs). Mean pairwise differences among species from the same genus for the COI region are: Cassowary (*Casuarius sp.*) 2.40% (37 sites, 2 species, 2 sequences, 1544 basepairs), Kiwi 1.62, 6.02 and 6.09% (25, 93 & 94 sites, 3 species, 3 sequences, 1544 basepairs), Rhea 7.12% (110 sites, 2 species, 3 sequences, 1544 basepairs). It is noteworthy that three putative Kiwi (Tokoeka: *Apteryx australis*) subspecies (phylogenetically discreet units) show 1.84, 2.37 and 2.76% difference among subspecies for a partial control region (14, 18 & 21 mean pairwise differences, 3 units, 12 sequences, 761 basepairs) [21]. A specimen identification request for the King Island Emu COI haplotypes on the Barcode of Life Data Systems v2.5 database [22], which holds a large selection of COI region DNA barcodes, returns a 100% probability of placement within the modern Emu species, compared to 88.03% specimen similarity with the next best match being the Cassowary.

Low variation in the control region is generally unexpected. Potential causes of this low DNA sequence diversity might include a genetic bottleneck in the ancestral emu population or slow evolutionary or mutation rates. However, other ratites and birds show rates that are quite fast when compared to other animals [23], [24]. A likely cause for the minor divergence between both taxa is a very recent isolation of the King Island population from the modern Emu population. This scenario is based on the hypothesis that the King Island Emu were only recently isolated due to sea level changes in the Bass Strait, as opposed to a founding emu lineage that diverged from modern Emu far earlier and has subsequently gone extinct on the mainland. Models of sea level change indicate that Tasmania, including King Island, was isolated from the Australian mainland around 14,000 years ago. Up to several thousand years later King Island was then separated from Tasmania (Figure 2) [25], [26]. This scenario would suggest that initially a King Island/Tasmanian Emu population was isolated from the mainland taxon (which corresponds with fossil emu from Tasmania showing a similar size to the modern Emu), after which the King Island and Tasmanian populations were separated. This in turn indicates that the Tasmanian Emu is probably as closely related to the modern Emu as is the King Island Emu, with both the King Island and Tasmanian Emu being more closely related to each other. Fossil emu show an average size, between that of the dwarf and modern Emu taxa. Hence, modern Emu can be regarded as a large or gigantic form. It is remarkable that a lineage of this same group again evolved to a smaller form, within a short time span, possibly due to insular dwarfism as a result of phenotypic plasticity [27].

**Figure 4:**
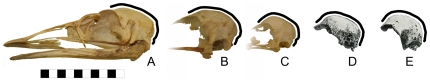
Comparison of the cranium contour in modern and King Island Emu. Several (partial) skulls from modern Emu are shown at different stages in their development: A – Adult, B – Immature-Adult, C – Juvenile. Two partial skulls are shown for the King Island Emu D & E [8]. The black lines indicate the contour of the upper/rear surface of the cranium. The adult and immature-adult modern Emu show a frontally flattened cranium, whereas King Island Emu show a more dome shaped cranium. Initially this difference was considered a species level difference, but juvenile modern emu show the same dome shaped cranium in both taxa and therefore appears not to be taxonomically significant.

The King Island Emu and the modern Emu show few morphological differences other than their significant difference in size. Additional traits that supposedly distinguish these taxa have previously been suggested to be plumage colour, the distal foramen of the tarsometatarsus, and the contour of the cranium. However, the distal foramen is known to be variable in the modern Emu showing particular diversity between juvenile and adult forms and is therefore taxonomically insignificant [10]. The same is true of the contour of the cranium, which is more dome-shaped in the King Island Emu but is in fact also seen in juvenile modern Emu (Figure 4). Due to their close genetic/evolutionary relationship and similar morphology it seems inappropriate to suggest that King Island Emu should be given species-status. Other terrestrial animals that are restricted to King Island are not typically considered endemic or different species, but rather subspecies or the same species with regard to their relatives living on Tasmania and/or mainland Australia. For example animals like the Echidna (*Tachyglossus aculeatus*), the common Brushtail (*Trichosurus vulpecula*) and Ringtail (*Pseudocheirus peregrinus*) Possum and the Black Tiger Snake (*Notechis ater*) are part of a Tasmania-wide subspecies, whereas the Spotted-tailed Quoll (*Dasyurus maculates*) and the Blotched Blue-tongued Lizard (*Tiliqua nigrolutea*) form part of (sub-) species that are also found on mainland Australia [28], [29]. Given the data presented here, subspecies status appears more appropriate for the King Island Emu in the form of *Dromaius novaehollandiae ater.* Just like the Quagga (*Equus quagga quagga*), one of the first species to have its ancient DNA sequenced, the King Island Emu proves to be an extinct subspecies on the basis of ancient DNA analyses, despite showing morphological diversification [30], [31]. This study also highlights the independence of processes governing morphological and neutral molecular evolution [32], [33]. King Island Emu show a significant reduction in size when compared to modern Emu, yet show little molecular diversification of mitochondrial loci. In contrast, recent studies have shown that the unique Tuatara of New Zealand (*Sphenodon* sp.) show a high molecular substitution rate in mitochondrial loci but little morphological diversification over millions of years [34]. Taken together these results suggest that rates of neutral molecular and morphological evolution are decoupled in both directions and either can evolve faster than the other. For example our work suggests that size and possibly melanism can evolve rapidly and thereby give the appearance of ‘distant’ relationships but the molecular data suggest the modern and King Island Emu shared a recent common ancestor with incomplete lineage sorting.

## Materials and Methods

### Extraction

DNA was extracted from five King Island Emu sub-fossil bones (Museum Victoria numbers B226 45, 50, 55 and 57, KI01-04 in this study respectively, B226 43 was excluded from further analysis after molecular damage proved too excessive) together with a mock extraction in a dedicated ancient DNA facility at Griffith University, Nathan, Australia. Positions 5457–5636 and 16108–16332 of the cytochrome *c* oxidase subunit I gene and control region, respectively, were independently replicated for KI02 and KI04 at the Alan Wilson Centre for Molecular Ecology and Evolution at Massey University, Albany, New Zealand. Approximately 200 mg of bone was incubated overnight in 3 ml of 0.5 M EDTA with 0.5 mg/ml Proteinase K and 0.1% Triton X-100. The solution was extracted with equal amounts of phenol and phenol/chloroform/isoamyl-alcohol (25∶24∶1) subsequently. The aqueous layer was incubated with 0.5 volumes of 7.5 M ammonium acetate, 2.5 volumes ethanol and 50 µl of linear polyacrylamide (LPA) and incubated at −20°C for 20 min. The DNA/LPA complex was centrifuged at 20,000× g for 15 min and the supernatant was washed with 200 µl of isopropanol and redissolved in 200 µl of ddH2O. Where required, DNeasy Blood & Tissue Kit (Qiagen) and Vivaspin Sample Concentrators (GE Healthcare) were used according to manufacturer's instructions to remove any PCR-inhibitors. DNA was extracted from the modern Emu blood samples using the former kit and in accordance with the manufacturer's instructions.

### Amplification

A multiplex PCR approach was used to amplify overlapping fragments of the complete mitochondrial control region (CR) and cytochrome *c* oxidase subunit I (COI) gene, a separate PCR amplified a fragment from the nuclear melanocortin 1 receptor (MC1R) gene (7×214 bp avg., 10×209 bp avg. and 1×95 bp amplicons respectively). The first stage 50 µl multiplex reaction contained 5 µl template, 1× PCR buffer, 1 mg/ml Bovine Serum Albumin, 2.5 mM MgCl2, 0.4 µM of each primer, 0.5 mM of each dNTP and 2 units of Platinum Taq (Invitrogen), thermocycling was as follows: 1 min 94°C, 40× (30 sec 94°C, 30 sec 55°C, 30 sec 72°C), 5 min 72°C. The odd primer mix contained: 5′-3′ (eCR1F: ACGGTCTGAAAAACCRTCG- eCR1R: GAATATGAGGTAAATATAAGTATGTACG); (eCR3F: TTCAGTGCTGTTACGGTCTAC- eCR3R: AGGAATGACCTCGACTTAGGA); (eCR5F: TAACCTTCAACGTACCCCC- eCR5R: GTGGAAATACCATAACCAGATG); (eCR7F: AACRCATCGTTAACACACATT- eCR7R: CTTCAGTGCCATGCTTTGATG); (eCOI1F: AGGACTACAGCCTAACGCTTA- eCOI1R: TGGTCATCTCCTAGTAGTGTT); (eCOI3F: GTGCTCCAGACATGGCATT- eCOI3R: GATGGAGGAAACACCAGCTA); (eCOI5F: TCCTACTATCGCTCCCAGT- eCOI5R: TTCCCTGCGTAATAAGTCAC); (eCOI7F: TCCGCTACCATAATCATCGC- eCOI7R: GAGAGGACATAATGGAAATGGG); (eCOI9F: ACCTTCTTCCCACAACACTTC- eCOI9R: ATGGATTCACTCAATGTTGGT); The even primer mix contained: 5′-3′ (eCR2F: CATTCAATATACGTACTATACCCAT- eCR2R: ATCCCGATTGACGAGCAG); (eCR4F: CCTGCCCACAACATGGT- eCR4R: TAAATTGTGAGCCTGCTGAC); (eCR6F: CATTCGGRCTCTGATGCAC- eCR6R: TGTAACTCCAGTACTGATGAC); (eCOI2F: CCTACTTATCCGTGCTGAACT- eCOI2R: GGTAAGAGTCAAAAGCTCATGTT); (eCOI4F: GGCTTCTGTAGATCTTGCCAT- eCOI4R: AGGTTTCGGTCTGTGAGGA); (eCOI6F: CCCAGGCTTTGGAATAATCTC- eCOI6R: TAGCTAATCAGCTGAATACCTTA); (eCOI8F: ATCGCCCTACATGATACATACTA- eCOI8R: TGAGTATCGTCGTGGTATTCC); (eCOI10F: AAAGTTGCCCAACCAGAACTA- eCOI10R: GAGGTTCGATTCCTTCCTTTC); keeping overlapping amplicons in separate reactions. The second stage 20 µl multiplex contained 1 µl template, 1× PCR buffer, 1 mg/ml Bovine Serum Albumin, 1.5 mM MgCl2, 0.4 µM of each primer (single pair), 0.2 mM of each dNTP and 1 unit of Platinum Taq, thermocycling was as follows: 1 min 94°C, 40× (30 sec 94°C, 30 sec 70°C, 30 sec 72°C), 5 min 72°C. The MC1R PCR contained 1 µl template, 1× PCR buffer, 1 mg/ml Bovine Serum Albumin, 1.5 mM MgCl2, 0.4 µM of each primer (5′ eMC1RF: TGCTGCCTGGCCGTCTCC- eMC1RR: TGGATCACCAGCACGCCGTG 3′), 0.2 mM of each dNTP and 1 unit of Platinum Taq, thermocycling was as follows: 1 min 94°C, 50× (30 sec 94°C, 30 sec 70°C, 30 sec 72°C), 5 min 72°C. DNA from the modern Emu specimens was amplified using the same protocol but with long range amplicons: eCR1F- eCR7R, eCOI1F-eCOI10R.

### Sequencing

The DNA was either isolated from a gel (in case of unspecific by-products) using the QIAquick Gel Extraction Kit (Qiagen) or cleaned with ExoSAP-IT (USB) according to manufacturer's instructions. The BigDye V3.1 (Applied Biosystems) kit was used according to manufacturer's instructions to sequence the DNA fragments. Sequences showing mononucleotide repeats were re-amplified with Phusion High-Fidelity DNA polymerase (Finnzymes) according to manufacturer's instructions [35]. Sequences are deposited in Genbank under accession numbers HQ910418-HQ910432.

### Computational

After assembly the DNA sequences were manually screened for errors (*i.e.* contamination and molecular damage) and re-sequenced accordingly (at least two independent subsequent PCRs per ambiguous amplicon). The sequences were aligned with publicly available Emu sequences for each region and a concatenated alignment was created for the control and COI regions. The Emu mitochondrial genome reference sequence NC_002784 showed a cytosine deletion at position 15,648 when compared with other emu sequences, this deletion was ignored for analyses as it is likely to be a sequencing error [35], the emu reference sequence was included in all subsequent analyses. Pairwise distances were calculated using MEGA 4 [36] as uncorrected *p*-distances between the groups using complete deletion for gaps. The haplotype network was constructed using TCS [37] with a 95% connection limit, no gaps were present in the alignment.
